# Ferroptosis-related genes as diagnostic markers for major depressive disorder and their correlations with immune infiltration

**DOI:** 10.3389/fmed.2023.1215180

**Published:** 2023-10-24

**Authors:** Jingjing Chen, Xiaolong Jiang, Xin Gao, Wen Wu, Zhengsheng Gu, Ge Yin, Rui Sun, Jiasi Li, Ruoru Wang, Hailing Zhang, Bingying Du, Xiaoying Bi

**Affiliations:** ^1^Department of Neurology, The First Affiliated Hospital of Naval Medical University, Shanghai, China; ^2^Department of Laboratory Animal Sciences, School of Basic Medicine, Naval Medical University, Shanghai, China

**Keywords:** major depression disorder, ferroptosis, immune infiltration, GEO database, diagnostic biomarkers

## Abstract

**Background:**

Major depression disorder (MDD) is a devastating neuropsychiatric disease, and one of the leading causes of suicide. Ferroptosis, an iron-dependent form of regulated cell death, plays a pivotal role in numerous diseases. The study aimed to construct and validate a gene signature for diagnosing MDD based on ferroptosis-related genes (FRGs) and further explore the biological functions of these genes in MDD.

**Methods:**

The datasets were downloaded from the Gene Expression Omnibus (GEO) database and FRGs were obtained from the FerrDb database and other literatures. Least absolute shrinkage and selection operator (LASSO) regression and stepwise logistic regression were performed to develop a gene signature. Receiver operating characteristic (ROC) curves were utilized to assess the diagnostic power of the signature. Gene ontology (GO) enrichment analysis was used to explore the biological roles of these diagnostic genes, and single sample gene set enrichment analysis (ssGSEA) algorithm was used to evaluate immune infiltration in MDD. Animal model of depression was constructed to validate the expression of the key genes.

**Results:**

Eleven differentially expressed FRGs were identified in MDD patients compared with healthy controls. A signature of three FRGs (ALOX15B, RPLP0, and HP) was constructed for diagnosis of MDD. Afterwards, ROC analysis confirmed the signature’s discriminative capacity (AUC = 0.783, 95% CI = 0.719–0.848). GO enrichment analysis revealed that the differentially expressed genes (DEGs) related to these three FRGs were mainly involved in immune response. Furthermore, spearman correlation analysis demonstrated that these three FRGs were associated with infiltrating immune cells. ALOX15B and HP were significantly upregulated and RPLP0 was significantly downregulated in peripheral blood of the lipopolysaccharide (LPS)-induced depressive model.

**Conclusion:**

Our results suggest that the novel FRG signature had a good diagnostic performance for MDD, and these three FRGs correlated with immune infiltration in MDD.

## 1. Introduction

Major depression disorder (MDD) is a devastating neuropsychiatric disease and has become one of the leading causes of suicide worldwide (1). Early diagnosis and appropriate treatment would undoubtedly reduce the mortality and improve the life quality of the affected individuals (2). However, to date, diagnosis of MDD mainly depends on subjective identification of symptoms cluster by neuropsychologists, leading to a high rate of misdiagnosis (3–5). Albeit many types of objective biomarkers have been explored for years, such as neurotransmitters, pro-inflammatory cytokines and small non-coding RNAs (6–8), none of these biomarkers have been clinically used, largely due to their limited diagnostic accuracy or inconsistent results (5, 9, 10). Therefore, it is of great clinical value to identify more valid and reliable biomarkers for MDD.

Ferroptosis is a recently discovered form of programmed cell death, marked by iron metabolism and lipid peroxidation. It has been proven to play a vital role in the survival of neural cells in several neurological diseases, such as stroke, Alzheimer’s disease and MDD (11). A few studies have reported changes of ferroptosis-related indicators in animal models of MDD (12, 13). However, the expression levels of ferroptosis-related genes (FRGs) in MDD patients, and whether they can be applied as diagnostic markers for MDD have not been elucidated.

It is well-known that immune response is implicated in the pathophysiology of MDD, including dysregulated actions of immune cells (14). Moreover, accumulating evidence indicated that ferroptosis could alert immune system, and ferroptosis is also impacted by immune cells in turn (15, 16). Thus, it is meaningful to evaluate the relationship between FRGs and immune cells in MDD.

In this study, based on the GEO database, differentially expressed FRGs were identified, and then they were selected to construct and validate a FRG gene signature for the diagnosis of MDD. Furthermore, the associations between FRGs and immune cell infiltration were also assessed to better understand and explore the immune mechanisms during the development of MDD.

## 2. Materials and methods

### 2.1. Data collection and processing

The microarray datasets used in the study were obtained from the GEO database.^1^ Totally, three datasets regarding MDD were collected (17, 18). The GSE98793 dataset contains blood samples from 128 MDD patients and 64 healthy controls (17). GSE76826 consists of blood samples from 20 MDD patients and 12 healthy controls (19). GSE53987 collects brain tissue of 17 MDD patients and 19 healthy controls from three brain regions, including hippocampus, prefrontal cortex, and striatum (20). Detailed information of these three datasets was summarized in Supplementary Table 1.

The GSE76826 dataset was downloaded in raw form. Raw files were submitted to background correction, and then normalized using the “limma” R package from R/Bioconductor software (21). The series matrix files of GSE98793 and GSE53987 were downloaded by the “GEOquery” R package. Notably, the samples of GSE98793 were tested in two batches. Thus, the “SVA” R package with the ComBat method was used to remove the batch effect (22). Probe IDs were converted to corresponding gene symbols according to annotation information provided in the GEO database.

### 2.2. Identification of differentially expressed ferroptosis-related genes

A total of 259 FRGs were retrieved from the FerrDb database (23).^2^ Moreover, 395 FRGs were collected from the previous literatures (24, 25). After deleting overlapping genes, a total of 476 FRGs were finally confirmed (Supplementary Table 2).

The GSE98793 dataset was used as the training set. The “limma” R package was used to perform significance analysis of the differentially expressed genes (DEGs) between MDD patients and healthy controls in GSE98793. The selection criteria were *P* < 0.05 and | log fold change| (| log FC|) >0.2.

The FRGs intersected with the DEGs, and then the differentially expressed FRGs (DE-FRGs) were identified. Volcano plots and boxplots were exhibited using the “ggplot2” R package. Heatmaps were drawn by the “heatmaps” R package. The correlation matrix analysis of the DE-FRGs was visualized using the “corrplot” R package.

### 2.3. Construction and validation of the diagnostic gene signature

To minimize the risk of overfitting, least absolute shrinkage and selection operator (LASSO) regression was conducted using the “glmnet” package in the GSE98793 dataset (26). Then, stepwise multivariate logistic regression was applied to construct a diagnostic signature. The diagnostic score was calculated according to the expression level of each gene and its corresponding regression coefficient. The formula was established as follows: score = $\sum_{i=1}^{n}\text{E}\,x\,p\,i\times \beta \,\text{i}$. Here, *n* represents the number of diagnostic genes, *Expi* represents the expression level of gene *i*, and β*i* represents the regression coefficient of gene *i* from the multivariate logistic regression.

Subsequently, receiver operating characteristic (ROC) curves with area under the curves (AUCs) were performed to assess the predictive power of the gene signature by the “pROC” R package. Bootstrap resampling method with 1,000 iterations of resampling was utilized for internal validation, and GSE76826 was employed as an external validation dataset using the same coefficients in the diagnostic signature.

### 2.4. Gene ontology enrichment analysis

The “Guilt-by-association” method was used to explore biological functions of those genes obtained from the above gene signature (27). The association between each diagnostic gene and DEGs from GSE98793 was examined by spearman correlation analysis. Based on DEGs associated with each diagnostic gene, gene ontology (GO) enrichment analysis was performed by the “clusterProfiler” package (28).

### 2.5. Protein–protein interaction network construction

To detect the potential relationships among the proteins by the DEGs from GSE98793, we used the Search Tool for the Retrieval of Interacting Genes (STRING) database, version 12.0 to construct a protein–protein Interaction (PPI) network; a combined score of >0.4 was set as the cut-off criterion of statistical significance. Cytoscape 3.9.1 was used to visualize the PPI network. Ten genes with the highest degree of connectivity were identified as hub genes using the CytoHubba plugin.

### 2.6. Immune infiltration analysis

The relative infiltration of 28 immune cell types in each sample of GSE98793 were estimated by the single sample gene set enrichment analysis (ssGSEA) with the “GSVA” R package (29, 30). Special feature gene panels of each immune cell subset were collected from previous researches (31, 32). The abundance of each immune cell type was shown by an enrichment score and visualized by heatmaps and boxplots. Spearman correlation analysis was performed to explore the association between infiltrating immune cells and each diagnostic gene.

### 2.7. Animal experiments

All procedures were approved by the Animal Care Committee of Naval Medical University and in accordance with the Animal Research Guidelines for the Care and Use of Laboratory Animals. ICR male mice (aged of 10–11 weeks, weighting 25–30 g) were obtained from the animal center at Naval Medical University. Before the onset of these experiments, 1 week was allowed for the mice to adapt to their new environments. All mice were housed under standard laboratory conditions (temperature 22 ± 1°C; humidity 52 ± 2%; 12 h day/night rhythm) with food and water available ad libitum.

Depression model was established with low dose of lipopolysaccharide (LPS) administration as previously described (33). In brief, LPS (from *Escherichia coli* 0111:B4, Sigma-Aldrich, cat#L2630) was dissolved in sterile saline and administered intraperitoneally (i.p.). The mice were randomly assigned to the vehicle control (0.9% saline) and the LPS group (0.8 mg/kg, i.p.) (33). Depressive behavioral tests were performed 24–30 h after LPS administration.

Tail suspension test (TST) and forced swimming test (FST) are widely used to assess depression-like behavior in rodent models (34, 35), TST was followed by FST in this study. TST was conducted according to our previous study (36), that is, mice were suspended with tails taped to the edge of a shelf that was 30 cm above a table for 6 min. After 2 min of adaptation, the immobility duration over the last 4 min were measured. FST was performed as previously described (37). Each mouse was individually placed into a Plexiglas cylinder (30 cm height by 20 cm diameter) containing 20 cm height of 25 ± 1°C water for 6 min. The total immobility time during the last 4 min was measured in a blinded manner. Besides, each mouse was ensured to be dried after each trial, and the water was changed between subjects.

After the final behavioral test, mice were anesthetized with sodium pentobarbital (75 mg/kg). Blood samples were collected from retro-orbital sinus and then centrifuged to obtain the serum. The serum levels of ALOX15B, RPLP0, and HP were measured using an enzyme-linked immunosorbent assay (ELISA) following the manufacturer’s instructions (Yanjin Biotechnology Co., Ltd., Shanghai, China).

### 2.8. Statistical analysis

All statistical analyses were performed by R software (version 4.0.2) and GraphPad Prism 7.0. Student’s *t*-test or Mann–Whitney *U* test was conducted to compare the differences in continuous variables as appropriate, whilst the Chi-squared test was used to compare the categorical variables. All significance tests were two-sided, and a *P* < 0.05 was considered as statistically significant.

## 3. Results

### 3.1. Identification of DE-FRGs between MDD patients and healthy controls

**FIGURE 1:**
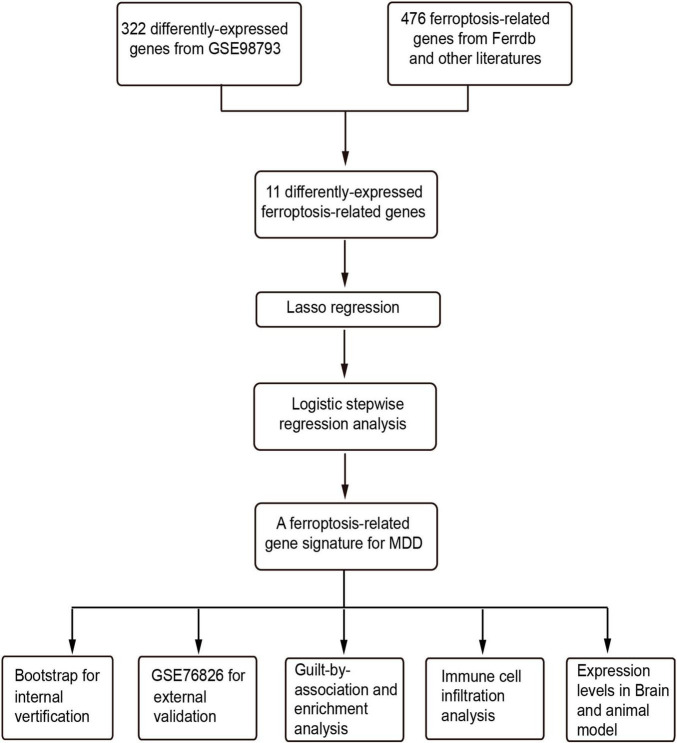
The flowchart of this study. LASSO, least absolute shrinkage and selection operator; GO, gene ontology.

**FIGURE 2:**
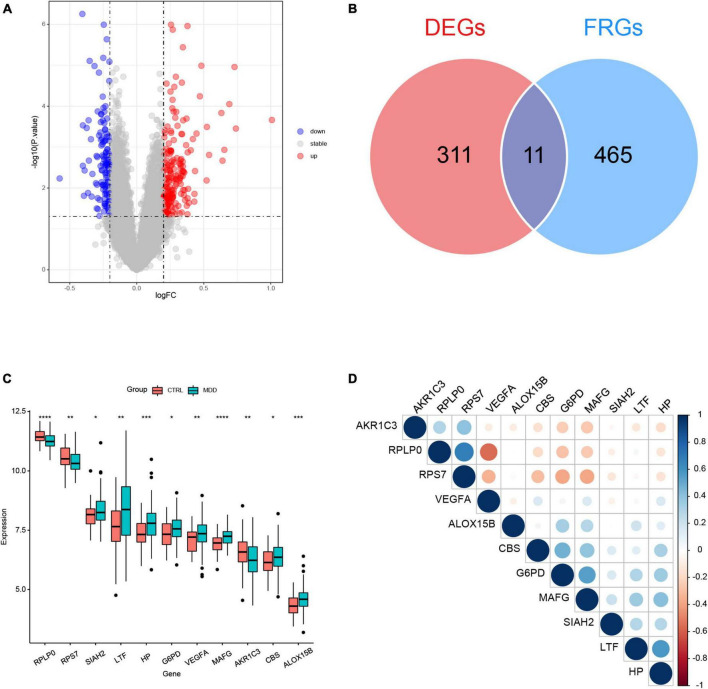
Identification of differentially expressed genes (DEGs) between major depression disorder (MDD) patients and healthy controls in GSE98793. **(A)** Volcano plot of the DEGs. **(B)** Venn plot showing the overlap between the DEGs and ferroptosis-related genes (FRGs). **(C)** Boxplot of the expression values of the eleven differentially expressed ferroptosis-related genes (DE-FRGs); *P < 0.05, **P < 0.01, ***P < 0.001, and ****P < 0.0001. **(D)** Correlation heatmap of the eleven DE-FRGs. The sizes of dots represent the strength of correlations, and the darker a dot is, the stronger a correlation is. Blue dots represent positive correlations, and red dots represent negative correlations.

The flowchart of this study was shown in Figure 1. The clinical and demographic characteristics of MDD patients and healthy individuals in GSE98793 were summarized in Table 1. In total, 322 DEGs were identified in the dataset GSE98793 based on | logFC| >0.2 and *P* < 0.05, of which 192 (59.6%) were upregulated and 130 (40.4%) were downregulated (Figure 2A). By intersecting 476 FRGs, 11 DE-FRGs were obtained, including CBS, VEGFA, MAFG, AKR1C3, G6PD, ALOX15B, SIAH2, HP, RPS7, RPLP0, and LTF (Figure 2B). The expressions of the eleven genes were shown in Figure 2C and Supplementary Figure 1.

**TABLE 1:** Clinical information of MDD patients and healthy controls in GSE98793.

	MDD patients (*n* = 128)	Healthy controls (*n* = 64)	*P*-value
Age (years, median [IQR])	52.7 [43.8, 62.0]	52.5 [43.9, 62.0]	0.985
Gender (*n*, M/F)	32/96	16/48	1

MDD, major depression disorder; M, male; F, female; IQR, inter quartile range.

### 3.2. Construction and validation of a ferroptosis-related gene signature for MDD

**FIGURE 3:**
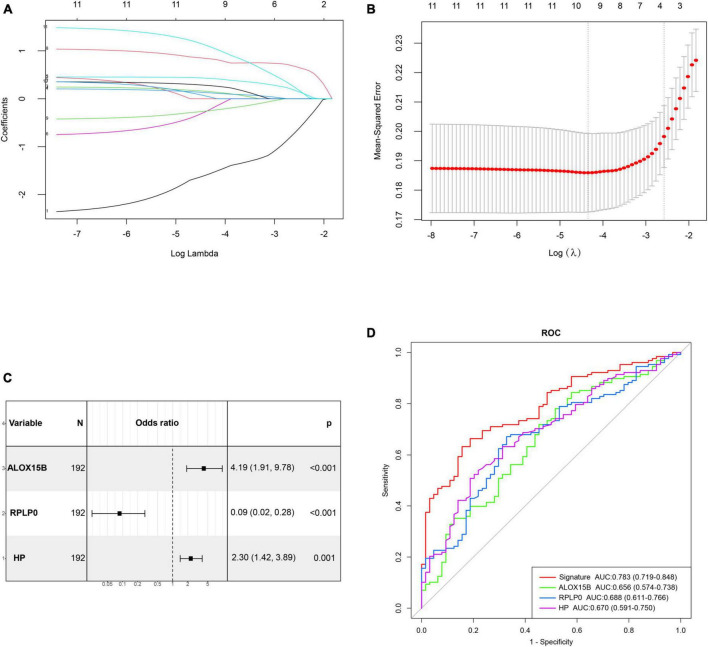
Construction and validation of a diagnostic ferroptosis-related gene signature for MDD. **(A,B)** LASSO regression analysis with lambda.1se as the best lambda. **(C)** Forest plot exhibiting independently diagnostic ferroptosis-related genes screened through stepwise multivariate logistic regression analysis. **(D)** ROC curves showing the diagnostic power of the gene signature.

The correlation matrix analysis of the eleven DE-FRGs was shown in Figure 2D. Some genes exhibited strong correlations with others. Thus, LASSO regression was used to solve the multicollinearity problem and to narrow down the DE-FRGs as candidate diagnostic genes. Based on lambda.1se, four DE-FRGs (ALOX15B, MAFG, HP, and RPLP0) were selected (Figures 3A, B).

Subsequently, age, gender and these four genes mentioned above were incorporated into stepwise multivariate logistic regression to establish the optimal diagnostic model for MDD, which resulted in a three-gene signature including ALOX15B, RPLP0, and HP. The results of stepwise logistic regression were shown in Figure 3C. Moreover, the diagnostic ability of these three genes were evaluated by ROC curves with an AUC of 0.656 (95% CI, 0.574–0.738) for ALOX15B, 0.688 (95% CI, 0.611–0.766) for RPLP0 and 0.670 (0.591–0.750) for HP, respectively (Figure 3D). Then, the diagnostic score was calculated as follows: score = (1.213 × expression level of ALOX15B) + (−2.145 × expression level of RPLP0) + (0.692 × expression level of HP). The result showed that the AUC of the diagnostic score reached 0.783 (95% CI, 0.719–0.848), indicating a high discriminative power for MDD.

The internal validation of the gene signature performed by bootstrap resampling demonstrated good discrimination with a bias-corrected AUC of 0.788 (95% CI, 0.712–0.849). Furthermore, the diagnostic efficiency of the gene signature was externally validated in another independent dataset GSE76826. The result showed an AUC of 0.713 (95% CI, 0.526–0.899), which also indicated a prominent diagnostic capacity (Supplementary Figure 2). The demographic features of MDD patients and healthy controls in GSE76826 were summarized in Supplementary Table 3.

### 3.3. Functional analysis of the diagnostic genes

**FIGURE 4:**
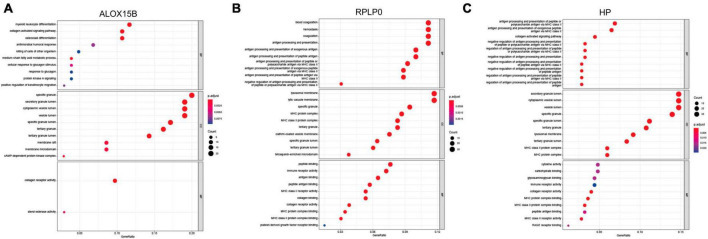
The biological roles of ALOX15B, RPLP0, and HP in MDD. **(A–C)** GO enrichment analyses of DEGs associated with the three FRGs, respectively. The larger the circle size, the more count of DEGs, and the color represents *P*-value, the redder the color, the smaller the value. BP, biological process; CC, cellular component; MF, molecular function.

To further detect biological roles of these three FRGs, “Guilt-by-association” method was used. In GSE98793, spearman correlation analysis showed that ALOX15B was significantly associated with 104 DEGs, RPLP0 with 249 DEGs, and HP with 239 DEGs (all *P*s < 0.05, Supplementary Tables 4–6). Moreover, GO enrichment analysis revealed that these three groups were enriched in immune response, immune cell activity and differentiation, suggesting that immune dysregulation plays an important role in MDD and ferroptosis may be correlated with immune response (Figures 4A–C).

### 3.4. Protein–protein interaction network construction

**FIGURE 5:**
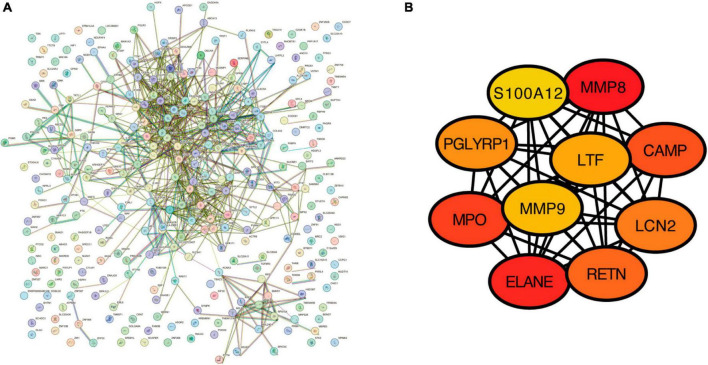
The construction of protein–protein interaction network. **(A)** Protein–protein interaction network for the proteins encoded by the DEGs using the STRING database. **(B)** The top 10 hub genes identified in the PPI network visualized using Cytoscape software.

The PPI network of the 322 DEGs has 290 nudes and 766 edges, the average node degree of the PPI network was 5.28, and average local clustering coefficient was 0.416, and the PPI enrichment *P*-value was <1e−16 (Figure 5A). The topological analysis of the PPI network identified ten hub genes, including MMP8, ELANE, MPO, CAMP, RETN, LCN2, PGLYRP1, LTF, MMP9, and S100A12 (Figure 5B).

### 3.5. Immune cell infiltration

**FIGURE 6:**
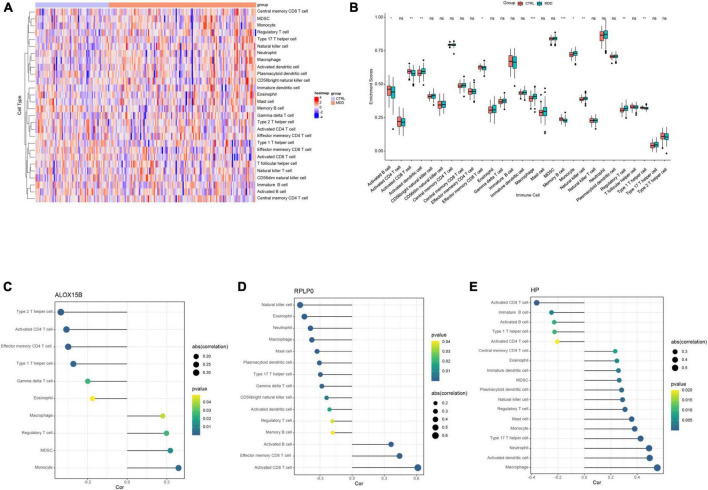
The results of immune cell infiltration analysis in GSE98793. **(A)** Heatmap of the relative abundance of the 28 immune cells. **(B)** Boxplot exhibiting the differences of the 28 immune cells between MDD patients and healthy controls. **(C–E)** Correlation between ALOX15B, RPLP0, and HP with infiltrating immune cells in MDD patients. *P < 0.05, **P < 0.01, ***P < 0.001, and *^ns^*no significance. The size of a dot represents the strength of a correlation, the stronger the correlation, the bigger the dot.

Based on the aforementioned GO enrichment results, the relative abundance of 28 immune infiltrating cells in GSE98793 was quantified by ssGSEA and was shown in Figure 6A. The enrichment scores of activated dendritic cells, macrophages, monocytes, natural killer cells, and regulator T cells in MDD patients were significantly higher than those in healthy controls, while the scores of activated B cell, activated CD8+ T cell, effector memory CD8+ T cell, memory B cell, and type 1 T helper cell were lower (all *P*s < 0.05, Figure 6B).

### 3.6. Correlations between diagnostic genes and infiltrating immune cells

Spearman correlation analysis was performed to explore the associations between infiltrating immune cells and these three abovementioned FRGs. The correlation analysis revealed that ALOX15B was positively associated with monocyte (*r* = 0.259, *P* = 0.003), MDSC (*r* = 0.218, *P* = 0.014), and macrophage (*r* = 0.180, *P* = 0.042), and negatively correlated with type 2 T helper cell (*r* = −0.334, *P* < 0.001), active CD4+ T cell (*r* = −0.306, *P* < 0.001), and gamma delta T cell (*r* = −0.199, *P* = 0.024) in MDD patients (Figure 6C and Supplementary Table 7). RPLP0 had a positive correlation with activated B cell (*r* = 0.364, *P* < 0.001), activated CD8+ T cell (*r* = 0.613, *P* < 0.001), and negative correlation with natural killer cell (*r* = −0.485, *P* < 0.001), eosinophil (*r* = −0.445, *P* < 0.001), neutrophil (*r* = −0.389, *P* < 0.001), and macrophage (*r* = −0.376, *P* < 0.001) (Figure 6D and Supplementary Table 8). HP was positively associated with macrophage (*r* = 0.553, *P* < 0.001), activated dendritic cell (*r* = 0.495, *P* < 0.001), neutrophil (*r* = 0.491, *P* < 0.001), monocyte (*r* = 0.382, *P* < 0.001) and negatively with activated CD8+ T cell (*r* = −0.361, *P* < 0.001) and type 1 T helper cell (*r* = −0.226, *P* = 0.010) (Figure 6E and Supplementary Table 9).

### 3.7. Expression levels of the diagnostic genes

**FIGURE 7:**
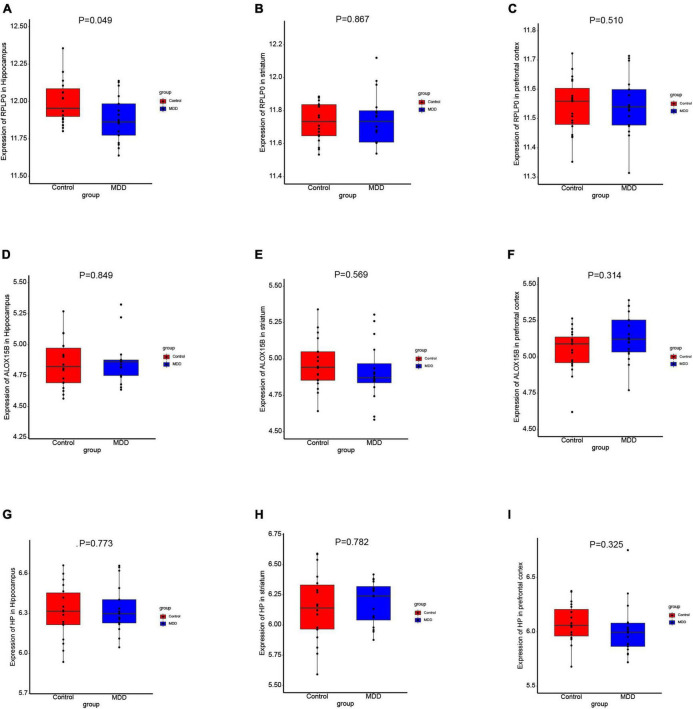
The expression levels of these diagnostic genes in brain. **(A–I)** Boxplots of the expression values of RPLP0, ALOX15B, and HP in the hippocampus (*n* = 35), prefrontal cortex (*n* = 36), and striatum (*n* = 34) of MDD patients and healthy controls in GSE53987.

**FIGURE 8:**
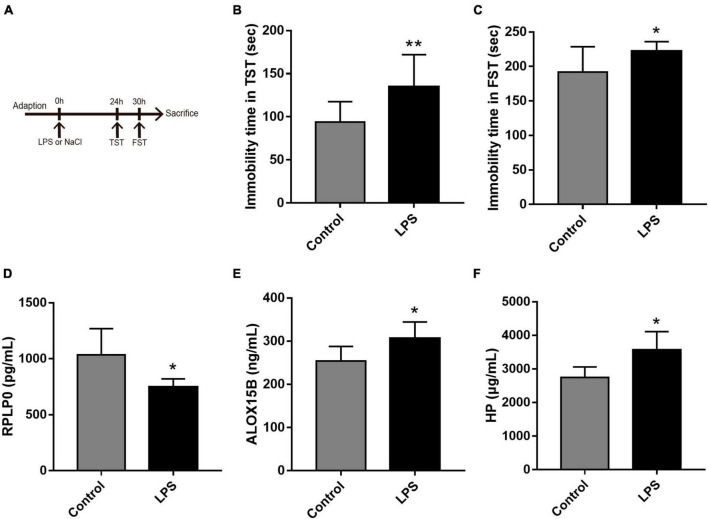
The experimental design and results in the LPS-induced depressive animal model. **(A)** The time schedule of the experiment. **(B,C)** Immobility time in the tail suspension test (TST) and in the forced swim test (FST, *n* = 10 per group). **(D–F)** The concentrations of ALOX15B, RPLP0, and HP in serum after the administration of LPS or saline (*n* = 5–6 per group). Data are expressed as means ± SEM. *P < 0.05 and **P < 0.01.

To further explore the expression values of ALOX15B, RPLP0, and HP in brain of MDD patients, GSE53987 was analyzed. The level of RPLP0 in the hippocampus was significantly lower than that in healthy controls (*P* < 0.05) (Figure 7A), while there was no significant difference of RPLP0 in the striatum and prefrontal cortex (Figures 7B, C). There was no difference of ALOX15B and HP in these three brain areas between these two groups (Figures 7D–I). To further demonstrate the expression levels of these genes in animal model, we established an acute depression mouse model with a low dosage of LPS injection (Figure 8A). Compared with the control group, the LPS group showed significantly longer immobility time in TST (*P* = 0.009, Figure 8B) and FST (*P* = 0.027, Figure 8C), indicating that the LPS group developed depressive-like behaviors 24–30 h post LPS administration. Then, we measured the concentrations of ALOX15B (also known as Alox8 in mice), RPLP0 and HP in our depression mouse model. The concentrations of ALOX15B and HP in LPS group showed a significant elevation (*P* = 0.045 and *P* = 0.014, respectively), and RPLP0 was significantly down-regulated compared with the control group (*P* = 0.018, Figures 8D–F).

## 4. Discussion

Major depression disorder is one of the common neuropsychiatric disorders, affecting approximately 17% of the population at some point in life (38). Identification of reliable and effective biomarkers is of great clinical value for MDD. Ferroptosis is a recently discovered iron-dependent type of regulated cell death, and FRGs have been reported as diagnostic and/or prognostic biomarkers in multiple types of tumors (39, 40). However, there is little research on FRGs in neuropsychiatric diseases. To our knowledge, this is by far the first study to explore the roles of FRGs in MDD. Here, we not only constructed and validated a three-gene signature (ALOX15B, RPLP0, and HP), which demonstrated good diagnostic power for MDD, but also for the first time studied the relationship between FRGs and immune cell infiltration in MDD patients. Moreover, we validated the expression value of these three genes in a LPS-induced depression model.

Several studies also reported some gene signatures for MDD. In the original study of GSE98793, Leday et al. identified 165 differentially expressed immune-related genes and got an AUC of 0.71 classifying MDD patients and controls based on the combination of the 165 genes (17). He et al. constructed a gene signature using four autophagy-related genes identified in their study and obtained a diagnostic AUC of 0.779 (41). Furthermore, Zhao et al. reported an optimal panel of 70 feature genes with the SVM classifier and yielded an AUC of 0.82 in discriminating MDD from healthy individuals (42). Moreover, the study of Papakostas et al. suggested that a panel of nine serum biomarkers can yield sensitivity and specificity of approximately 90 and 80% (43). In contrast, considering the relatively small number of biomarkers and the high discrimination of the gene signature (AUC = 0.783) in our study, it seems that these three FRGs may have greater potential for clinical practice.

Of these three FRGs, RPLP0, and HP have been reported to be associated with MDD (44, 45). Ribosomal protein lateral stalk subunit P0 (RPLP0) is a gene expressed in the central nervous system and encodes a ribosomal protein that is a component of the 60S subunit (45, 46). Previous studies showed that downregulation of RPLP0 could result in accumulation of reactive oxygen species through activating MAPK1/ERK2 signaling pathway, thereafter, decrease cell proliferation and lead to cell cycle arrest (47). As we found in this study, several studies also reported that RPLP0 was downregulated in brain and blood samples of MDD patients, suggesting that it may play a crucial role in the development of MDD (45, 46, 48).

HP encodes haptoglobin, a plasma protein that binds free hemoglobin and protects against heme-driven oxidative stress. Haptoglobin also plays an essential role in regulating immune response at the acute phase of diseases (49). MDD is accompanied by systemic immune activation described by higher levels of pro-inflammatory cytokines and positive acute-phase proteins (14). Mounting studies have reported elevated levels of haptoglobin in MDD patients and the levels of haptoglobin were significantly related to the numbers of circulating immune cells, such as leukocytes, monocytes, and neutrophils, which are consistent with the findings in this study (44, 50). Of note, a further HP variant is the protein named “zonulin,” a tight-junction protein modulating both gut and blood–brain barriers. Increased circulating levels of zonulin indicates the loss of integrity of these protective layers, resulting in increased passage to the bloodstream and the brain of irritative materials, such as pro-inflammatory stimuli and bacterial endotoxins (51). Zonulin has also been reported to be overexpressed in plasma of MDD patients, indicating that the microbiota-gut-brain axis may underlie the development of MDD (52, 53).

Arachidonate 15-lipoxygenase type B (ALOX15B, known as Alox8 in mice), encodes a non-heme-iron-containing enzyme, a member of the lipoxygenase family that catalyzes the deoxygenation of polyunsaturated fatty acid in arachidonic acid metabolism (54). It is constitutively expressed in macrophages, and its expression can be further enhanced by the stimulus of LPS, IL-4, and hypoxia (55). To date, the biological functions of ALOX15B have not been fully understood. Proinflammatory activation of macrophages could lead to activation of ALOX15B, and reduction of ALOX15B decreases inflammation and lipid accumulation in macrophages, suggesting an active proinflammatory role of ALOX15B (56, 57). ALOX15B and its splice variants are also identified in human epithelial cells of prostate, skin, esophagus, and cornea, and possess multiple biological functions, including inhibition of cell-cycle progress and proliferation, induction of a senescence-like phenotype, and inhibition of tumor progression (54). However, the roles of ALOX15B in MDD has not been previously reported. In our study, ALOX15B was overexpressed in blood samples of MDD, and we infer it may be associated with proinflammatory activation of macrophages. Furthermore, Jäckle et al. reported that ALOX15B was highly expressed at the rim of slowly expanding lesions in the brains of multiple sclerosis, which was concordant with the activated microglia/macrophages in situ (58). In our study, however, we did not observe increased levels of ALOX15B in brain samples of MDD patients. Therefore, more experimental studies are needed to detect the expression of ALOX15B in brain, and to explore its role in MDD.

It is well known that MDD is accompanied by systematic immune activation, including dysregulation of immune cell numbers. Several immune cells, such as macrophages, monocytes, and dendritic cells have been reported to be increased in blood of MDD patients (14, 59). Meanwhile, there also exists evidence that depression is accompanied by immunosuppression. For example, some studies reported decreased lymphoproliferative response of T cells or reduced number of T helper cells (60, 61). In this study, we utilized ssGSEA method, a widely used algorithm, to conduct a comprehensive evaluation of immune cell infiltration in MDD, and our results are consistent with the findings above.

Ferroptosis can impact immune cells in two different ways. On the one hand, immune cells themselves can undergo ferroptosis. On the other hand, ferroptotic cells can be recognized by immune cells and then trigger subsequent immune response (16). Thus, we analyzed the correlation between ALOX15B, RPLP0, and HP with infiltrating immune cells, and found that these three FRGs were significantly associated with immune cells, indicating an interaction between ferroptosis and immune response in MDD.

Several limitations should be emphasized when interpreting the findings in the current study. Firstly, this is a second mining of previously published datasets, and more longitudinal studies are needed to elucidate the role of FRGs in MDD. Secondly, the sample size of the external validation was relatively small, and more large-scale clinical studies are required to further validate the diagnostic power of the signature identified in the study. Thirdly, whether the signature based on these three FRGs is specific to MDD is still unknown, and its role in differentiating MDD from other conditions, such as bipolar disorder experiencing an acute major depressive episode, remains to be elucidated. Lastly, immune cell infiltration in MDD patients was estimated by ssGSEA, and these predictions need to be confirmed by further studies.

## 5. Conclusion

In conclusion, the current study for the first time provided insight into FRGs in MDD. A FRG signature was established for the diagnosis of MDD, which demonstrated good discrimination. These three FRGs (ALOX15B, RPLP0, and HP) identified in this study may represent diagnostic markers for MDD. Furthermore, immune infiltration analysis indicated that ferroptosis may be involved in immune response in MDD. These findings extend our knowledge concerning FRGs in MDD patients.

## Data availability statement

The original contributions presented in this study are included in this article/Supplementary material, further inquiries can be directed to the corresponding authors.

## Ethics statement

The animal study was approved by the First Affiliated Hospital of Naval Medical University. The study was conducted in accordance with the local legislation and institutional requirements.

## Author contributions

XB and BD conceived and designed the study. JC, XJ, and XG performed the data analysis and wrote the original draft. WW, ZG, GY, and RS collected the data. JL, RW, and HZ reviewed and revised the manuscript. All authors contributed to the article and approved the submitted version.

## Footnotes

1
https://www.ncbi.nlm.nih.gov/geo/

2
http://www.zhounan.org/ferrdb/legacy/index.html
