# Supplementary material for: Ferroptosis-related genes as diagnostic markers for major depressive disorder and their correlations with immune infiltration
Source: Front Med (Lausanne). 2023 Oct 24;10:1215180. doi: 10.3389/fmed.2023.1215180 (PMC10627962; doi:10.3389/fmed.2023.1215180)
Supplement: Supplementary file 1 [file Data_Sheet_1.docx]

Supplementary Material

**Ferroptosis-Related Genes as Diagnostic Markers for Major Depressive Disorder and Their Correlations with Immune Infiltration**

Jingjing Chen^1^†, Xiaolong Jiang^2^†, Xin Gao^1^†, Wen Wu^1^, Zhengsheng Gu^1^, Ge Yin^1^, Rui Sun^1^, Jiasi Li^1^, Ruoru Wang^1^, Hailing Zhang^1^, Bingying Du^1^* and Xiaoying Bi^1^*

*** Correspondence:** Xiaoying Bi: [bixiaoying2013@163.com](mailto:bixiaoying2013@163.com); Bingying Du: 15800614142@163.com

# Supplementary Figures and Tables

## Supplementary Figures


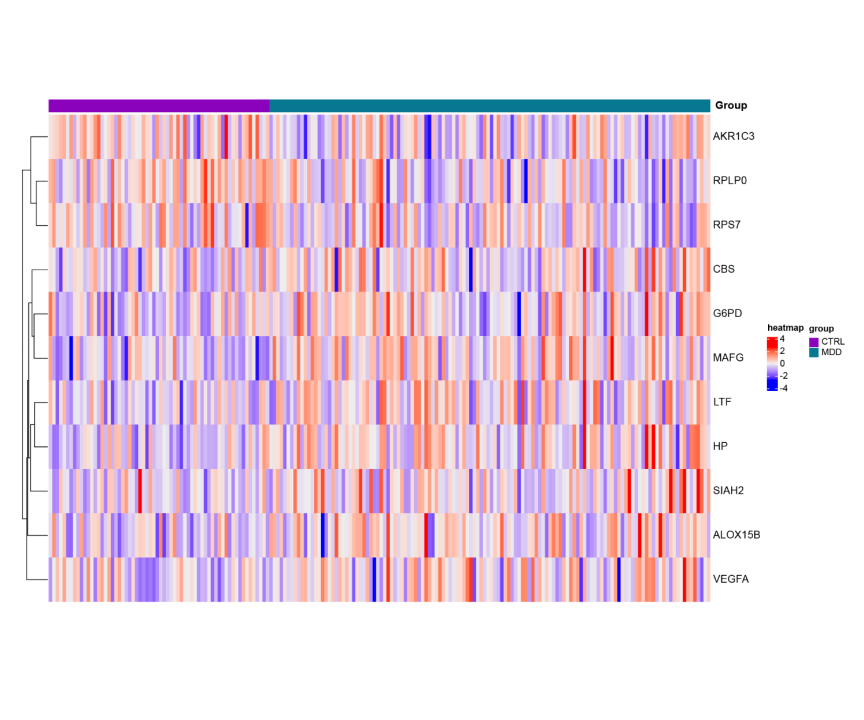


**Supplementary Figure 1.** The heatmap of the expression levels of the eleven differentially expressed ferroptosis-related genes in GSE98793.


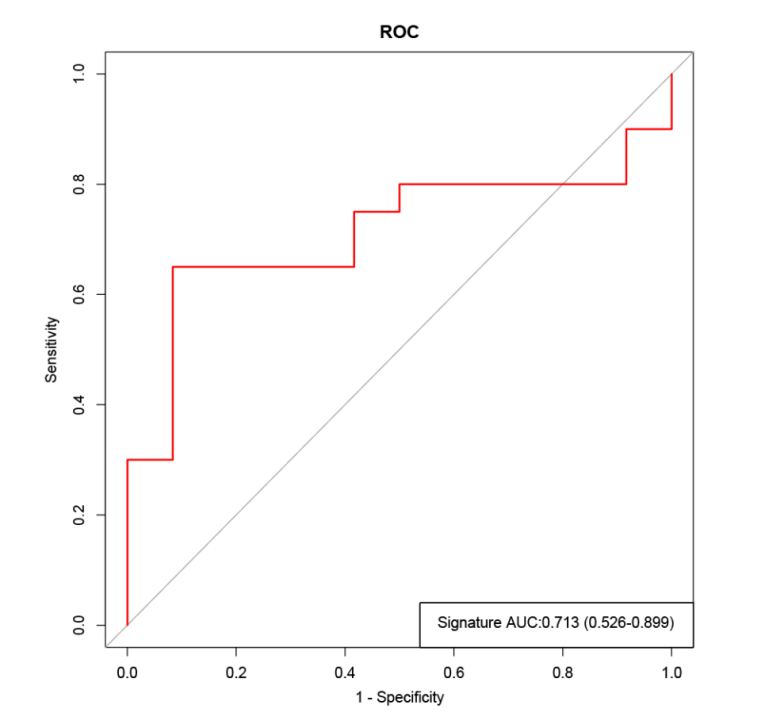


**Supplementary Figure 2.** Receiver operating characteristic (ROC) curves showed the predictive efficiency of the gene signature in GSE76826.

## Supplementary Tables

### Table 1. Description of the datasets in this study.

| **Accession** | **Platform** | **Species** | **Sample** | **Reference (PMID)** |
| --- | --- | --- | --- | --- |
| GSE98793 | GPL570 | Homo sapiens | whole blood | 28688579 |
| GSE76826 | GPL17077 | Homo sapiens | whole blood | 26926397 |
| GSE53987 | GPL570 | Homo sapiens | Brain tissue | 31123247 |

### Table 2. The 476 Ferroptosis-related genes included in this study

Please see the excel file named “Table 2.XLS”

### Table 3. Clinical information of MDD patients and healthy controls in GSE76826.

|  | **MDD patients (n=20)** | **Healthy controls**  **(n=12)** | ***P*** |
| --- | --- | --- | --- |
| Age (years, median [IQR]) | 75.5[64.8, 81.2] | 59.5 [55.5, 68.8] | 0.033 |
| Gender (n, M/F) | 9/11 | 5/7 | 1 |

MDD, major depression disorder; M, male; F, female; IQR, Inter Quantile Range.

### 1.2.4 Table 4. The 104 DEGs associated with ALOX15B in GSE98793.

Please see the excel file named “Table 4.xlsx”.

### Table 5. The 249 DEGs associated with RPLP0 in GSE98793.

Please see the excel file named “Table 5.xls”.

### Table 6. The 239 DEGs associated with HP in GSE98793.

Please see the excel file named “Table 6.xls”.

### Table 7. The infiltrating immune cells associated with ALOX15B in MDD patients.

| **Cell type** | **r** | **P** |
| --- | --- | --- |
| Type 2 T helper cell | -0.334 | <0.001 |
| Activated CD4 T cell | -0.306 | <0.001 |
| Effector memeory CD4 T cell | -0.297 | 0.001 |
| Type 1 T helper cell | -0.271 | 0.002 |
| Monocyte | 0.259 | 0.003 |
| MDSC | 0.218 | 0.014 |
| Gamma delta T cell | -0.199 | 0.024 |
| Regulatory T cell | 0.199 | 0.025 |
| Macrophage | 0.180 | 0.042 |
| Eosinophil | -0.174 | 0.049 |

### Table 8. The infiltrating immune cells associated with RPLP0 in MDD patients.

| **Cell type** | **r** | **P** |
| --- | --- | --- |
| Activated CD8 T cell | 0.613 | <0.001 |
| Natural killer cell | -0.485 | <0.001 |
| Eosinophil | -0.445 | <0.001 |
| Effector memeory CD8 T cell | 0.443 | <0.001 |
| Neutrophil | -0.389 | <0.001 |
| Macrophage | -0.376 | <0.001 |
| Activated B cell | 0.364 | <0.001 |
| Mast cell | -0.327 | <0.001 |
| Plasmacytoid dendritic cell | -0.306 | <0.001 |
| Type 17 T helper cell | -0.296 | 0.001 |
| Gamma delta T cell | -0.283 | 0.001 |
| CD56bright natural killer cell | -0.238 | 0.007 |
| Activated dendritic cell | -0.211 | 0.017 |
| Regulatory T cell | -0.184 | 0.037 |
| Memory B cell | -0.181 | 0.041 |

### Table 9. The infiltrating immune cells associated with HP in MDD patients.

| **Cell type** | **r** | **P** |
| --- | --- | --- |
| Macrophage | 0.553 | <0.001 |
| Activated dendritic cell | 0.495 | <0.001 |
| Neutrophil | 0.491 | <0.001 |
| Type 17 T helper cell | 0.426 | <0.001 |
| Monocyte | 0.382 | <0.001 |
| Activated CD8 T cell | -0.361 | <0.001 |
| Mast cell | 0.359 | <0.001 |
| Regulatory T cell | 0.308 | <0.001 |
| Natural killer cell | 0.290 | 0.001 |
| Plasmacytoid dendritic cell | 0.283 | 0.001 |
| MDSC | 0.264 | 0.003 |
| Immature dendritic cell | 0.259 | 0.003 |
| Immature B cell | -0.249 | 0.005 |
| Eosinophil | 0.246 | 0.005 |
| Central memory CD8 T cell | 0.234 | 0.008 |
| Activated B cell | -0.229 | 0.009 |
| Type 1 T helper cell | -0.226 | 0.010 |
| Activated CD4 T cell | -0.205 | 0.020 |
